# Evolving sunscreen-use prevalence, patterns and perceptions from an online survey of digitally active Africans

**DOI:** 10.1093/skinhd/vzag061

**Published:** 2026-05-07

**Authors:** Olufolakemi Cole-Adeife, Chinazo Onyema, Shakirat Gold-Olufadi, Nokubonga Khoza, Ehiaghe Anaba, Erere Otrofanowei, Utseoritsejor Tuedon, Ayesha Akinkugbe

**Affiliations:** Dermatology Unit, Department of Medicine, Lagos State University Teaching Hospital, Lagos, Nigeria; Keck School of Medicine, University of Southern California, Los Angeles, CA, USA; Department of Medicine, Brookdale University Hospital and Medical Centre, Brooklyn, NY, USA; Private Practice, Illovo, Sandton, South Africa; Dermatology Unit, Department of Medicine, Lagos State University Teaching Hospital/Lagos State University College of Medicine, Lagos, Nigeria; Dermatology Unit, Department of Medicine, Lagos University Teaching Hospital/College of Medicine, University of Lagos, Lagos, Nigeria; Department of Public Health, University of Wolverhampton, Wolverhampton, UK; Dermatology Unit, Department of Medicine, Lagos University Teaching Hospital/College of Medicine, University of Lagos, Lagos, Nigeria

## Abstract

This study explores, via a prospective cross-sectional online survey, sunscreen-use patterns and perceptions among digitally active people from sub-Saharan Africa. The survey reveals that 64% of respondents use sunscreen, primarily to prevent photoageing and hyperpigmentation, with broad-spectrum SPF >30 products the most commonly used. Fifty-six percent report reduced hyperpigmentation and sunburn with use. This study reports increased sunscreen use and knowledge of perceived benefits compared with previous African studies; however, knowledge gaps and cosmetic concerns (whitish casts and oiliness) are commonly reported.

Dear Editor, Individuals with richly pigmented skin have some natural photoprotection against ultraviolet (UV) radiation (UVR) due to higher eumelanin content and more efficient DNA repair mechanisms, which significantly reduces skin cancer incidence compared with those with lighter skin phototypes.^1,2^ However, this protection is insufficient against photoageing and UVR-associated hyperpigmentation.^1,2^

Uneven skin tone, postinflammatory hyperpigmentation and melasma, all exacerbated by UV exposure, are some of the most common concerns in people with skin of colour and significantly affect their quality of life.^1–3^ Hyperpigmentation is also a major motivator for skin bleaching, a harmful practice with severe health consequences.^4^ Recent studies have demonstrated the benefits of sunscreen use in managing hyperpigmentation and photoageing in people with darker skin tones, which could make it useful for discouraging skin bleaching.^1,2,5^ However, sunscreen use is reportedly low among people with darker skin types, due to the perception that darker skin offers adequate UV pro­tection.^1,2,5^ Additionally, limited awareness of sunscreen’s benefits beyond skin cancer prevention, cosmetic compatibility and affordability may contribute to low use among African individuals.^5–8^

The growing influence of social media trends on skincare habits may have increased global awareness and use of sunscreen.^9,10^ Yet, data on sunscreen-use patterns among digitally active people from sub-Saharan Africa are sparse. This study aimed to explore sunscreen-use patterns and perceptions among a cohort of individuals from sub-Saharan Africa who use popular social media platforms.

A preliminary cross-sectional study was conducted using a digital survey on Google Forms and distributed via Twitter (now X), Instagram, Facebook and WhatsApp from June to October 2022. Eligibility (inclusion) criteria included sub-Saharan African nationality, active social media use, age 16–75 years and residing within or outside Africa. Exclusion criteria included non-African nationalities. The minimum sample size was 385, based on a proportion of 0.5 for maximum variability, a 95% confidence level and 5% margin of error.^6,8^ The survey, conducted in English and French, was piloted with 20 respondents. It covered demographics, outdoor activity, sunscreen use, UVA and UVB protection, perceived benefits and adverse effects, some dermatological concerns and self-assessed Fitzpatrick skin type, based on image references.

Data were analysed using SPSS version 28 (IBM, Armonk, NY, USA) and Stata version 17 (StataCorp, Cary, NC, USA). Descriptive statistics summarized demographics, sunscreen-use patterns and perceptions. Logistic regression evaluated predictors of use, adjusting for age group (16–30 years, 31–50 years, ≥51 years), African region, residence within Africa, Fitzpatrick skin type, hyperpigmentation and skin-lightening product use, with adjusted odds ratios and 95% confidence intervals.

The study had 751 respondents; most respondents were women (*n* = 650; 86.6%) and aged 21–50 years (*n* = 676; 90.0%). Most had a tertiary education (*n* = 724/751; 96.4%) and 57.3% (*n* = 430/751) had postgraduate degrees. Respondents were predominantly Nigerian or South African, with a minority from other countries (Table 1); 98.5% (*n* = 740/751) were English-speaking and 89.5% (*n* = 672/751) resided in Africa. The majority of respondents (*n* = 692/751; 92.1%) self-identified as having Fitzpatrick skin types V and VI, 7.2% (*n* = 54/751) as having skin types III and IV, and 0.6% (*n* = 5/751) as having skin types I and II. Two-thirds of respondents spent more time indoors (*n* = 499/751; 66.4%), while 10.1% (*n* = 76/751) spent more time outdoors.

**Table 1:** Summary of respondent (*n* = 751) demographics, sunscreen-use patterns and perceptions

Demographic/characteristic, *n* (%)	
Age (years), mean (SD)	37.8 (10.1)
Sex	
Female	650 (86.6)
Male	99 (13.2)
Not disclosed	2 (0.27)
Nationality	
Nigeria	486 (64.7)
South Africa	175 (23.3)
Other^a^	90 (12.0)
Sunscreen use (*n* = 484)	
Sunscreen SPF >30	402 (83.1)
Broad UVA + UVB sunscreen use	328 (67.8)
Once-daily sunscreen application	333 (68.8)
Two to four times daily application	100 (20.7)
Pea-sized amount on the face	155 (32.0)
Two fingers on the face	231 (47.7)
Three fingers on the face	74 (15.3)
Reasons for sunscreen use (*n* = 484)	
Protection from sun damage	425 (87.8)
Prevention of hyperpigmentation	258 (53.3)
Antiageing	216 (44.6)
Popular opinion/media	112 (23.1)
Dermatologist/aesthetician recommended	85 (17.6)
Observed benefits of sunscreen use (*n* = 484)	
Reduced hyperpigmentation	269 (55.6)
Less sunburn	235 (48.6)
Fewer breakouts	87 (18.0)
No obvious benefit	88 (18.2)
Adverse effects experienced with sunscreen use (*n* = 484)	
Greyish or whitish cast	118 (24.4)
Eye stinging	111 (22.9)
Excessive oiliness	111 (22.9)
Excessive sweating	53 (11.0)
Irritation or breakouts	52 (10.7)
Hyperpigmentation	34 (7.0)
Current skin concerns	
Hyperpigmentation (dark spots, sunburn, melasma)	358 (47.7)
Pimples (acne)	234 (31.2)
Postacne scars	200 (26.6)
Stretch marks	148 (19.7)
‘Sensitive skin’	111 (14.8)
‘Eczema’ (tinea versicolor)	44 (5.9)
Factors associated with lower sunscreen use, aOR (95% CI), *P*-value	
Male sex (vs female)	0.14 (0.08–0.23), <0.001
Age 31–50 years (vs 16–30 years)	0.47 (0.32–0.70), <0.001
More time outdoors (>10 h vs >1–5 h)	0.28 (0.10–0.78), 0.02
More time outdoors (5–10 h vs >1–5 h)	0.62 (0.40–0.99), 0.04
West Africans (vs South Africans)	2.89 (1.92–4.34), <0.001
Factors associated with higher sunscreen use, aOR (95% CI), *P*-value	
FST I–III vs FST V and VI	7.36 (0.83–64.91), 0.07
History of skin lightening vs none	1.70 (0.83–3.50), 0.15
Hyperpigmentation (vs none)	0.80 (0.40–1.61), 0.53

Data are presented as *n* (%) unless otherwise stated. aOR, adjusted odds ratio; CI, confidence interval; FST, Fitzpatrick skin type; SPF, sun protection factor; UV, ultraviolet. ^a^Ghana, Cameroon, Gambia, Benin, Madagascar and Kenya.

Sunscreen use was reported by 64.4% (*n* = 484/751; 459 women, 25 men) of respondents, with 58.5% (*n* = 283/484) starting use within the last 3 years and 41.5% (*n* = 201/484) having used it for >3 years. Most used sunscreen with a sun protection factor (SPF) ≥30 (*n* = 402/484; 83.1%) and 67.8% (*n* = 328/484) used broad-spectrum formulas. Once-daily application (*n* = 333/484; 68.8%) and ‘two-finger’ quantities (*n* = 231/484; 47.7%) were common, but one-third of users (*n* = 155/484; 32.0%) applied only a pea-sized amount (Table 1).

Primary motivations for sunscreen use included sun protection, prevention of hyperpigmentation, antiageing goals, popular opinion and dermatologists’ recommendations. Common reported benefits included reduced hyperpigmentation (*n* = 269/484; 55.6%) and sunburn (*n* = 235/484; 48.6%), with 18.2% (*n* = 88/484) reporting no obvious benefit. Adverse effects, such as a whitish cast, eye irritation and oiliness, were commonly reported (see Table 1). Most participants (*n* = 558/751; 74.3%) believed that sunscreen is necessary for darker skin, 8.1% (*n* = 61/751) did not and 17.6% (*n* = 132/751) were unsure.

Multivariate analysis showed that sunscreen use was significantly lower among men, those aged 31–50 years and outdoor dwellers, but slightly higher among individuals with Fitzpatrick skin phototypes I–III and those who use skin-lightening products. Regionally, people from South Africa had higher odds of sunscreen use than individuals from West Africa. No significant difference was found between African residents and those living abroad (Table 1).

This study reports a higher prevalence of sunscreen use than in previous African studies (which focused on Nigerian traders and South African dermatology patients), where sunscreen knowledge and use were much lower (10–20%).^6–8^ This contrast may be due to the predominance of highly educated, digitally active women in this cohort, a demographic not typically represented in African research. However, with increasing globalization and wider internet coverage, more Africans are turning to digital and social media for skin health information.^9,10^ This population is more aware of the harmful effects of skin bleaching and often pursues international best practices for managing hyperpigmentation, which include photoprotection.^1,2,5^ The over-representation of Nigerian and South African respondents may be due to their larger populations, better internet access and the survey’s dissemination via the authors’ social media networks.

In this study, most participants reported using broad-spectrum high-SPF sunscreens, showing good knowledge of appropriate sunscreens. Sun protection, hyperpigmentation and premature ageing concerns were primary motivations for use, indicating a shift away from a historical reliance on skin-lightening products.^4^ However, many users applied sunscreen once daily and used insufficient amounts, signifying knowledge gaps on appropriate use. Nearly a quarter of participants cited popular opinion as the reason for using sunscreen, further emphasizing the role of media in sunscreen-use behaviour and the importance of dermatologists’ presence on these platforms.^9,10^

Most sunscreen users observed a reduction in hyperpigmentation and sunburn, but almost one-fifth reported no benefit. In contrast with previous studies of populations of skin of colour, the majority of participants in our study believed sunscreen use was important for darker skin.^1,5^ However, despite higher sunscreen use, cosmetic compatibility was a concern, with adverse effects such as a whitish cast, oiliness and irritation reported, consistent with previous studies in populations of skin of colour.^1,2,5^ Physical UV filters such as zinc oxide and titanium dioxide are more likely to cause a whitish cast, but they are particularly beneficial as they also block visible light, which contributes to hyperpigmentation in darker skin.^1,2,5^ Appropriately tinted physical sunscreens may improve compatibility for individuals with darker skin tones.^1,2,5^

There was higher sunscreen use among women than men, probably due to the traditionally lower male interest in aesthetic concerns, which may also explain the few male respondents. Higher sunscreen use and less time spent outdoors in younger respondents could be attributed to the influence of digital media and occupational differences.^1,9,10^ Higher sunscreen use among South Africans aligns with previous studies and may reflect South Africa’s wider diversity in skin tones and races, as well as greater sunscreen access and awareness.^8^

Study limitations include a skew towards highly educated women and English speakers, primarily from Nigeria and South Africa, which limits generalizability. The online survey may have excluded nondigital populations, although previous studies have documented sunscreen use in these groups.^6–8^ The cross-­sectional design and self-reported data introduce recall and reporting biases. Additionally, cultural beliefs and sunscreen affordability were not explored. Despite these limitations, this study provides insights into evolving sunscreen use among digitally active Africans. Further research on photoprotection practices in African populations is needed, with a larger, more representative and gender-balanced cohort.

In conclusion, this study highlights a higher prevalence of sunscreen use among digitally active individuals from sub-Saharan Africa, with primary motivations for use being the prevention of hyperpigmentation and photoageing. Further research and the development of sunscreens tailored to darker skin tones and tropical climates are recommended, particularly to manage hyperpigmentation and photoageing, and to tackle skin bleaching in African populations.

## Data Availability

The dataset for this study is publicly available at https://figshare.com/s/444e48d247cf72c54871.
